# Pharmacological Effects of JTT-551, a Novel Protein Tyrosine Phosphatase 1B Inhibitor, in Diet-Induced Obesity Mice

**DOI:** 10.1155/2014/680348

**Published:** 2014-05-29

**Authors:** Makoto Ito, Sumiaki Fukuda, Shohei Sakata, Hisayo Morinaga, Takeshi Ohta

**Affiliations:** Japan Tobacco Inc., Central Pharmaceutical Research Institute, 1-1 Murasaki-cho, Takatsuki, Osaka 569-1125, Japan

## Abstract

Protein tyrosine phosphatase 1B (PTP1B) is a negative regulator of leptin signaling as well as insulin signaling. JTT-551 is a new PTP1B inhibitor, which is reported to improve glucose metabolism by enhancement of insulin signaling. We have evaluated an antiobesity effect of JTT-551 using diet-induced obesity (DIO) mice. A single administration of JTT-551 was provided to DIO mice with or without leptin, and DIO mice were given food containing JTT-551 for six weeks. A single administration of JTT-551 with leptin treatment enhanced the food inhibition and the signal transducer and activator of transcription 3 (STAT3) phosphorylation in hypothalamus. Moreover, chronic administration of JTT-551 showed an antiobesity effect and an improvement of glucose and lipid metabolism in DIO mice. JTT-551 shows an antiobesity effect possibly by enhancement of leptin signaling and could be useful in the treatment of type 2 diabetes and obesity.

## 1. Introduction

The prevalence of obesity continues to increase rapidly worldwide. Body weight is normally maintained within a narrow range by a balance between energy intake and expenditure. When energy intake exceeds energy expenditure, excess energy is stored as triglyceride in adipose tissue, resulting in weight gain. Obesity is an important risk factor for type 2 diabetes, cardiovascular disease, and the metabolic syndrome. Effective antiobesity therapies are urgently needed [1–3].

Leptin, a hormone secreted by adipocytes, decreases body weight both by suppressing appetite and by increasing energy expenditure [4–6]. The brain, particularly the hypothalamus, integrates leptin and various other metabolic signals to regulate energy homeostasis and body weight by controlling both behavior and metabolic responses [7–9]. Genetic deficiency of leptin or functional leptin receptors also results in obesity and obesity-associated metabolic diseases in both animals and humans. Leptin administration decreases body weight and fat mass [10–12]; however, most obese individuals exhibit elevated circulating leptin levels and are less responsive to exogenously administrated leptin, consistent with a leptin resistance [13, 14].

Protein tyrosine phosphatase 1B (PTP1B) has been implicated in the negative regulation of the signaling pathway that phosphorylates the tyrosine residue. It is reported that PTP1B knockout mice exhibit increased insulin and leptin sensitivity and are resistant to high-fat diet-induced obesity (DIO) [15, 16]. DIO rats have a marked increase in PTP1B protein expression in the hypothalamus. It was recently reported that neuronal PTP1B knockout mice are hypersensitive to leptin and have reduced body weight and adiposity and increased energy expenditure [17, 18]. Treatment of DIO rats with PTP1B antisense oligonucleotide i.c.v. resulted in a decreased food intake, reduced body weight, and reduced adiposity [19]. These results suggest that PTP1B may play a pivotal role in the leptin resistance associated with obesity. Specific PTP1B inhibitors may thus be therapeutically beneficial in obesity as well as in type 2 diabetes.

JTT-551 is a novel PTP1B inhibitor, which is under development as an antidiabetic drug. JTT-551 increases the insulin-stimulated glucose uptake in L6 rat skeletal myoblasts (L6 cells), and single administration enhanced insulin receptor phosphorylation in liver. JTT-551 improves glucose metabolism by enhancement of insulin signaling [20].

In the present study, we evaluated the pharmacological profiles, especially the enhancement effect of leptin signaling, of JTT-551* in vivo*, and examined whether the compound could be useful as an antiobesity agent.

## 2. Materials and Methods

### 2.1. Chemicals

JTT-551 was synthesized by Japan Tobacco Inc., Central Pharmaceutical Research Institute (Osaka, Japan).

### 2.2. Animals

All the experiments received prior approval from the Committee for the Humane Care and Use of Animals of Biological/Pharmacological Research Laboratories, Central Pharmaceutical Research Institute, Japan Tobacco Inc., in accordance with the Japanese Law on Humane Treatment and Management of Animals.

Male six-week-old C57BL/6J mice were purchased from Charles River Japan, Inc. (Yokohama, Japan). Animals were housed in a climate-controlled room (temperature 23 ± 3°C, humidity 55 ± 15%, and 12 h lighting cycle) and allowed free access to diet and water.

### 2.3. Acute Effect on DIO Mice

Seven-week-old C57BL/6J mice were provided with 35% fat diet (Oriental Yeast Co., Osaka, Japan) (Table 1)* ad libitum*. A single oral administration of JTT-551 100 mg/kg was provided to 12-week-old male DIO mice that had been fasting overnight and then leptin solution 10 mg/kg intraperitoneal administration 1 h before feeding. Feeding was resumed immediately after dosing and the food was weighed at 2, 4, 8, and 24 h. Cumulative food intake was calculated from difference in the weight from that before feeding. Calorie intake was determined under the following provisions: fat, 9 kcal/g; carbohydrate, 4 kcal/g; protein, 4 kcal/g.

Moreover, JTT-551 100 mg/kg was provided to 13-week-old male DIO mice that had been fasting overnight and then leptin solution 10 mg/kg intraperitoneal administration 1 h before feeding. The hypothalamus was removed at 2 h after feeding. The hypothalamus was homogenized and insoluble material was removed by centrifugation. Supernatants were separated using SDS polyacrylamide gel electrophoresis and immunoblotting as previously described [20]. Membranes were probed with antibodies for total and phosphorylated STAT3 (Santa Cruz Biotechnology, CA, USA). Protein phosphorylation was calculated as the ratio of phosphorylated-to-total protein expression.

### 2.4. Chronic Effect on DIO Mice

Eight-week-old DIO mice were given 10 or 100 mg/kg food containing JTT-551 for six weeks. Body weight and food consumption were measured every week. In fed or fasting DIO mice at six weeks after JTT-551 treatment, blood samples were collected from orbital venous plexus and the blood glucose, triglyceride (TG), and total cholesterol (TC) levels were measured using commercial kits (Roche Diagnostics, Basel, Switzerland) and an automatic analyzer (HITACHI 7170S; Hitachi, Tokyo, Japan). Blood insulin and leptin levels were measured with a rat enzyme-linked immunosorbent assay (ELISA) kit (Morinaga Institute of Biological Science, Yokohama, Japan).

### 2.5. Statistical Analysis

Results of body weight, cumulative calorie intake, and blood chemistry values were expressed as the mean ± standard deviation (SD). Statistical analysis of mean values was performed using Dunnett's *t*-test (two-tailed). Differences were defined as significant at *P* < 0.05.

## 3. Results

### 3.1. Acute Effect on DIO Mice

**Figure 1:**
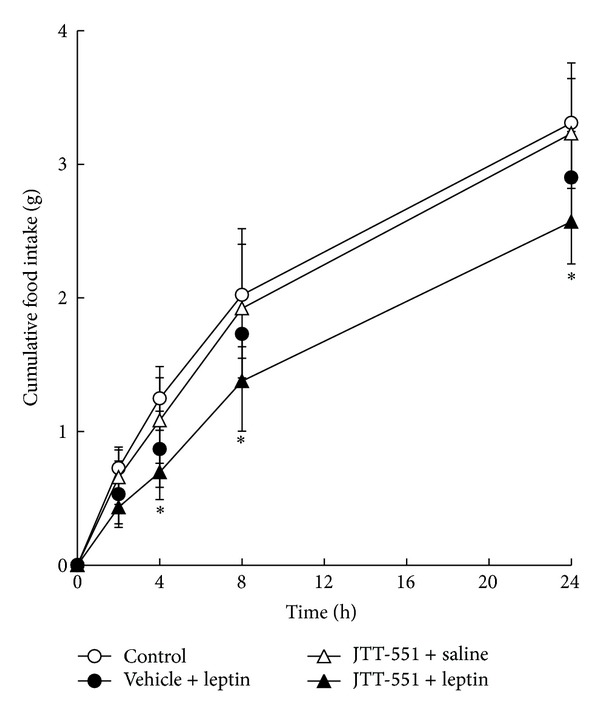
Enhancement effect of JTT-551 on leptin suppressed calorie intake in DIO mice. Data represent mean ± SD (*n* = 5).  *P < 0.05: significantly different from the control by Dunnett's test (two-tailed).

The results for food intake in DIO mice are shown in Figure 1. In leptin administration group (leptin group), food intake was reduced compared with that in vehicle (0.5% MC) administration control group (control group) from 2 h after feeding. In JTT-551 administration group without leptin treatment (JTT-551 group), food intake was not reduced. In JTT-551 with leptin administration group (JTT-551 + leptin group), food intake was significantly reduced compared with leptin group from 4 h after feeding.

**Figure 2:**
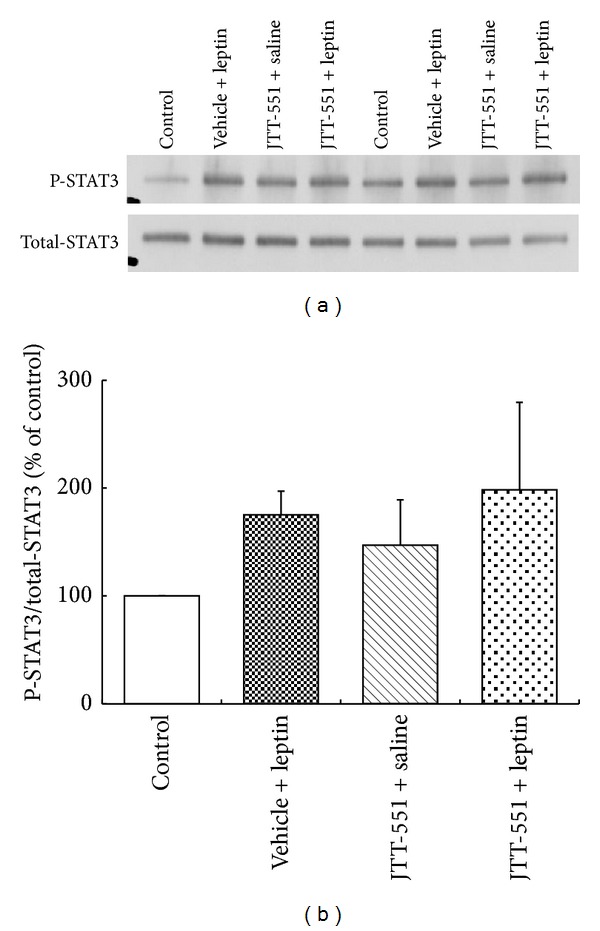
Enhancement effects of JTT-551 on STAT3 phosphorylation in the hypothalamus of DIO mice. The detected bands (typical bands) are shown in (a) and the phosphorylation-STAT3/STAT3 in each group in (b). The intensity of the STAT3 phosphorylation was calculated as the ratio of the density of phosphorylation-STAT3 to the density of STAT3.

The results of western blot analysis are shown in Figure 2. The detected bands (typical bands) are shown in Figure 2(a) and the phosphorylated STAT3/STAT3 in each group in Figure 2(b). The STAT3 phosphorylation in the hypothalamus after administration of leptin and/or JTT-551 was increased compared with that in control group. In JTT-551 + leptin group, the STAT3 phosphorylation was more enhanced than in single administration groups.

### 3.2. Chronic Effect on DIO Mice

**Figure 3:**
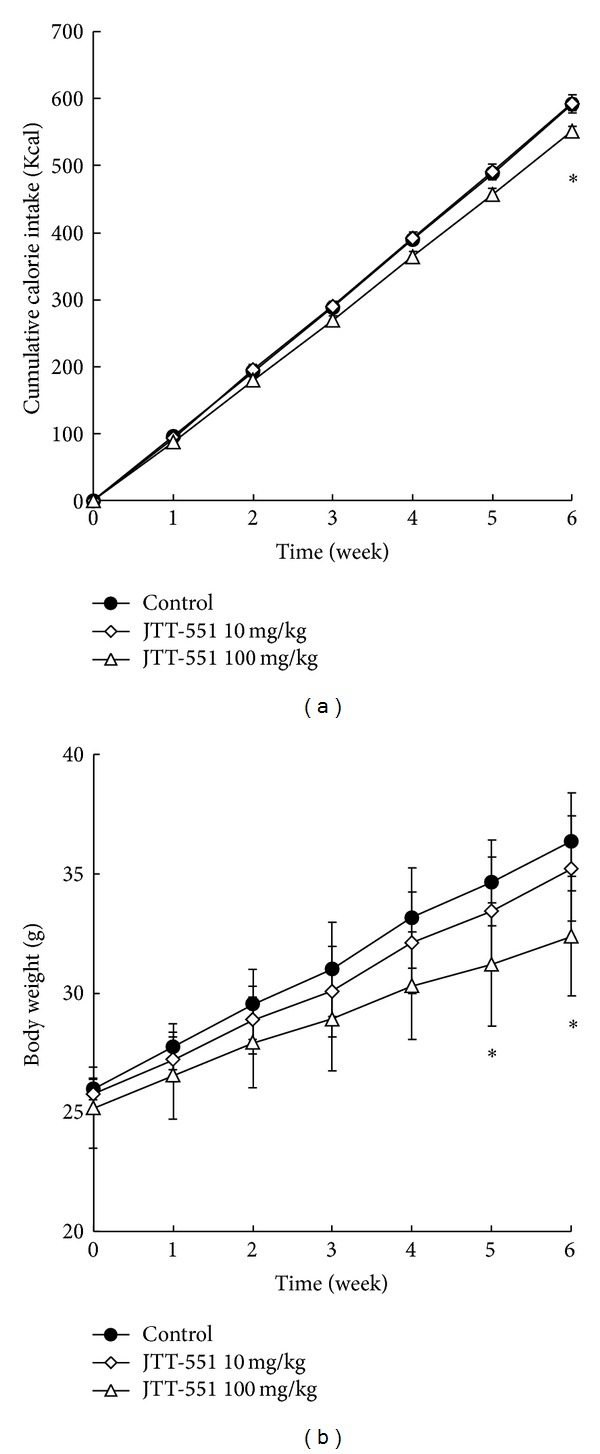
Effect of JTT-551 on cumulative calorie intake and body weight in DIO mice. DIO mice were given 10 or 100 mg/kg food containing JTT-551 for six weeks. Data represent mean ± SD (*n* = 5).  *P < 0.05: significantly different from the control by Dunnett's test (two-tailed).

Effects of JTT-551 on the cumulative food intake and body weight are shown in Figure 3. In the JTT-551 100 mg/kg group, the cumulative calorie intake tended to decrease from two weeks after treatment (control: 193.3 ± 7.6 Kcal and JTT-551 100 mg/kg: 183.8 ± 14.2 Kcal) and was significantly decreased from six weeks after treatment (control: 591.8 ± 21.8 Kcal and JTT-551 100 mg/kg: 560.7 ± 28.6 Kcal) (Figure 3(a)). The body weight in JTT-551 treatment tended to decrease dose-dependently (control: 36.4 ± 2.1 g, JTT-551 10 mg/kg: 35.2 ± 2.2 g, and JTT-551 100 mg/kg: 32.5 ± 2.3 g, at six weeks after treatment); the decreases in JTT-551 100 mg/kg group were significant from five to six weeks after treatment (Figure 3(b)).

**Figure 4:**
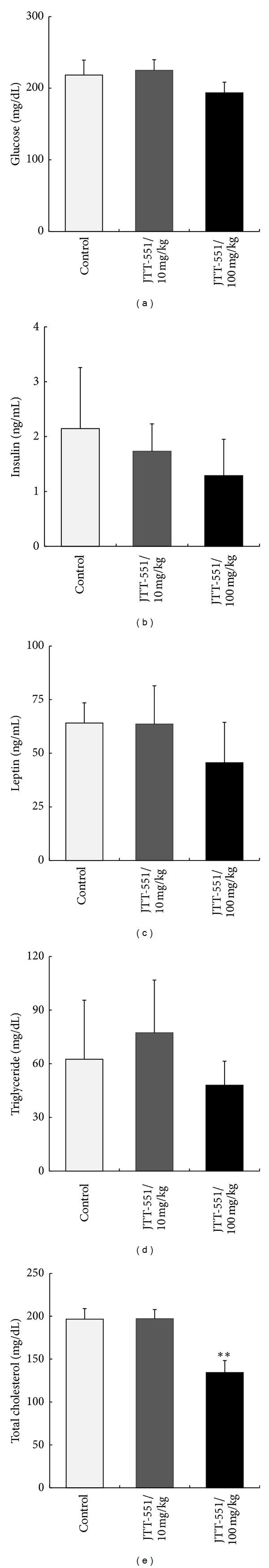
Effects of JTT-551 on blood glucose (a), insulin (b), leptin (c), triglyceride (d), and total cholesterol levels (e) in fed DIO mice. DIO mice were given 10 or 100 mg/kg food containing JTT-551 for six weeks. Data represent mean ± SD (*n* = 6).  **P < 0.01: significantly different from the control by Dunnett's test (two-tailed).

**Figure 5:**
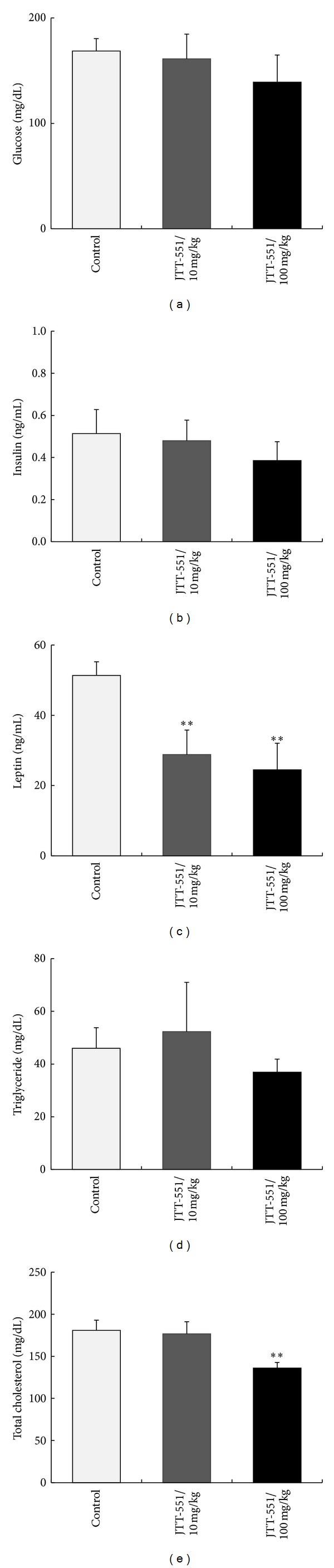
Effects of JTT-551 on blood glucose (a), insulin (b), leptin (c), triglyceride (d), and total cholesterol levels (e) in fasting DIO mice. DIO mice were given 10 or 100 mg/kg food containing JTT-551 for six weeks. Data represent mean ± SD (*n* = 6).  **P < 0.01: significantly different from the control by Dunnett's test (two-tailed).

Effects of JTT-551 on the blood chemistry values in six weeks after treatment are shown in Figures 4 and 5. The fed blood glucose level was not decreased (Figure 4(a)), but the fasting glucose level at JTT-551 100 mg/kg tended to decrease (control: 169 ± 12 mg/dL and JTT-551 100 mg/kg: 139 ± 26 mg/dL) (Figure 5(a)). The insulin levels in both fed and fasting mice tended to decrease, but not significantly (Figures 4(b) and 5(b)). The leptin levels in both fed and fasting mice tended to decrease dose-dependently, and those levels at JTT-551 treatment were significantly decreased (control: 51.3 ± 3.9 ng/mL, JTT-551 10 mg/kg: 28.8 ± 7.0 ng/mL, and JTT-551 100 mg/kg: 24.5 ± 7.5 ng/mL, in fasting mice) (Figures 4(c) and 5(c)). The TG levels in both fed and fasting mice tended to decrease, but not significantly (Figures 4(d) and 5(d)). The TC levels in both fed and fasting mice tended to decrease dose-dependently, and those levels at 100 mg/kg treatment were significantly decreased (control: 196.6 ± 12.4 mg/dL and JTT-551 100 mg/kg: 134.5 ± 13.9 mg/dL, in fed mice) (Figures 4(e) and 5(e)).

## 4. Discussion

PTP1B is a 50-KD cytosolic tyrosine dephosphorylase consisting of 435 amino acids which is ubiquitously expressed in organs throughout the body. It is well known that PTP1B dephosphorylates both phosphorylated insulin receptor (IR) *β* subunit and phosphorylated IR substrate, to negatively regulate insulin signal transmission [21, 22]. On the other hand, it is reported that PTP1B is concerned with negative regulation of leptin signal transmission, to dephosphorylate phosphorylated STAT3 [17, 18]. In a recent study, mice lacking the PTP1B were protected from diet-induced obesity and were hypersensitive to leptin. Neuronal PTP1B KO mice especially showed increased leptin signaling in the hypothalamus and had reduced feeding, weight, and adiposity and increased energy expenditure [15, 16]. This suggests that PTP1B is a key regulator of the leptin signal transmission. PTP1B is a negative regulator of leptin signal, in which the PTP1B inhibits Janus kinase 2 (JAK2)/STAT3 phosphorylation. The inhibition of PTP1B might induce an enhancement of leptin sensitivity. In this study, we investigated an antiobesity effect of JTT-551, which has been developed as a novel PTP1B inhibitor.

Inhibition of food intake in DIO mice was observed in leptin group. In JTT-551 + leptin group, the food intake inhibition was more strongly observed than in leptin group (Figure 1). JTT-551 showed an enhancement of food intake inhibition with leptin treatment. Furthermore, analysis of leptin signal with JTT-551 treatment was examined in DIO mice. Leptin stimulated the phosphorylation of STAT3 in hypothalamus. Also, JTT-551 enhanced the phosphorylation of STAT3 in leptin treatment (Figure 2). The food intake inhibition with JTT-551 might be caused by an enhancement of leptin signal. Leptin signal in the hypothalamus by binding to Ob-Rb to activate the tyrosine kinase JAK2 and the activated JAK2 phosphorylates itself and residues Tyr985 and Tyr1138 within the Ob-Rb cytoplasmic tail [23, 24]. Phosphorylated Tyr985 recruits the tyrosine phosphatase Shp2, resulting in leptin-evoked activation of extracellular signal-regulated kinase (Erk). Moreover, Tyr1138 recruits and activates the transcription factor STAT3, and the phosphorylated STAT3 is translocated into the nucleus and transcribed to various leptin target genes. In examination of genetic models, it is reported that leptin injection activated STAT3 in the hypothalamus of ob/ob mice and the wild mice but not db/db mice [23]. Since PTP1B dephosphorylates the phosphorylated JAK2 with insulin stimulation and inhibits the phosphorylation of STAT3 [17, 18], it is considered that JTT-551 enhanced the leptin signal via an enhancement of phosphorylation of STAT3 in DIO mice.

Obese-related leptin resistance and hyperleptinemia induce promotion of obesity, glucose and lipid metabolic abnormality, and hypertension. Leptin therapy did not show an efficacy for those diseases, and one of the reasons is considered to be a deterioration of leptin signal. Since the blood leptin levels in DIO mice were decreased by JTT-551 treatment (Figures 4(c) and 5(c)), leptin resistance might be improved by an inhibition of PTP1B. Furthermore, chronic administration of JTT-551 showed an antiobesity effect (Figure 3). With an antiobesity effect, long-term treatment with JTT-551 improved lipid disorder and tended to improve glucose metabolic abnormality (Figures 4 and 5). Pharmacological effect of JTT-551 is considered to be induced by the enhancement of insulin and leptin signals. However, the cumulative calorie intake in JTT-551 100 mg/kg group was significantly decreased in the late chronic phase, at six weeks after treatment (Figure 3(a)). Since the chronic administration of JTT-551 100 mg/kg may act as a feeding deterrent and induce the reduction of body weight, it is necessary to examine carefully the mechanism of an antiobesity effect with JTT-551 treatment in further study.

JTT-551 showed a blood glucose reduction and an improvement of insulin resistance at 10 mg/kg in ob/ob mice and a decrease of hemoglobin A_1*c*_ (Hb A_1*c*_) level at 30 mg/kg in db/db mice [20]. In our preliminary and present studies, an improvement of leptin signal in hypothalamus of DIO mice was observed at 100 mg/kg (Figures 1 and 2). An effective dose in leptin signal was higher than that in insulin signal. The reason for this might be a matter of brain penetration of JTT-551.

JTT-551, a novel developed PTP1B inhibitor, shows not only an improvement of glucose metabolism but also an antiobesity effect possibly by enhancement of leptin signaling and could be useful in the treatment of type 2 diabetes mellitus and obesity.

## Figures and Tables

**Table 1:** Composition of the experimental diet.

% (w/w)	35% fat diet
Lard	35.000
Cornstarch	7.308
Casein	28.810
Granulated sugar	14.410
Cellulose	7.200
AIN93M mineral mix	5.040
AIN93 vitamin mix	1.440
L-cystine	0.430
Choline bitartrate	0.360
Butylhydroquinone	0.002
